# Integrated drought monitoring framework for Eswatini applying standardised precipitation index and normalised difference vegetation index

**DOI:** 10.4102/jamba.v12i1.749

**Published:** 2020-12-14

**Authors:** Daniel H. Mlenga, Andries J. Jordaan

**Affiliations:** 1 Disaster Management Training and Education Centre for Africa, Faculty of Natural and Agricultural Sciences, University of the Free State, Bloemfontein, South Afrcia

**Keywords:** coordination, drought, drought monitoring, early warning, normalised difference vegetation index, preparedness, standard precipitation index

## Abstract

In the last decade, Eswatini has been affected by moderate to severe droughts, leading to huge impacts on the economic, environmental and societal sectors. The frequency and magnitude of drought have also increased, emphasising on the importance of drought monitoring. In view of the impacts of drought, it is of critical importance to monitor drought in near real-time and provide early warning information to stakeholders. The challenge however is the fragmentation of climatic data collection, the lack of agreed indicators and the poor coordination amongst institutions resulting in drought management being reactive, or ‘crisis management’ approach. A paradigm shift to a more risk reduction approach is therefore required to enable cost-effective and timely response to drought disasters. The capacity to monitor and predict the drought attributes (onset, frequency, duration and severity) is fundamental for spatiotemporal (drought) monitoring. Based on a review of country and regional networks, this research developed an integrated drought monitoring and early warning framework for Eswatini. The framework incorporated an early warning response trigger threshold derived from an integration of the standardised precipitation index and normalised difference vegetation index. The adoption of the framework allows for early warning and early action to mitigate the consequences of drought disasters. Drought preparedness and risk mitigation will help lower the eventual drought relief costs, protect food security and reduce the humanitarian impact on the population.

## Introduction

Drought is one of the most detrimental natural hazards causing adverse effects to social and ecological systems (Hao & AghaKouchak 2014). The insidious natural hazard is affecting the livelihoods of millions of people worldwide in many different ways, specifically 200 million people living in southern Africa, causing billions of dollars in loss annually, especially for the farming communities (FAO 2004). Droughts are becoming more frequent and severe in many countries of sub-Saharan Africa, causing huge damages to humanity, the environment and the economy (Masih et al. 2014). In the last decade, Eswatini has been affected by moderate to severe droughts, leading to huge impacts on the economic, environmental and societal sectors (Sheffield & Wood 2012; Smith & Katz 2013). The frequency and magnitude of drought have also increased, emphasising on the importance of drought monitoring. The dependence of Eswatini’s economy on rain-fed agriculture emphasises the importance of drought monitoring and early warning for decision-making.

According to Emergency Events Database (EM-DAT 2018), during the last century, southern Africa in general and Eswatini in particular have been characterised by an increase in frequency of droughts. Recorded drought years in the region include 1982–1983, 1987–1988, 1991–1992, 1994–1995, 1997–1998, 2002–2003 (Covele & Sannier 2005), 2005–2006, 2007–2008, 2009–2010, 2012–2013, 2015–2016 (EM-DAT 2018). Drought in Eswatini has almost followed a similar pattern as in the whole of southern Africa. Droughts have impacted the country differently in space and time (Swaziland National Vulnerability Assessment Committee [SNVAC] 2004, 2006, 2007, 2008, 2016). The impact of drought in Eswatini can be severe; this is because almost 70% of the population rely on rain-fed agriculture (SNVAC 2015) and over 40% of the country fall in the Lowveld agro ecological region which receives an annual average rainfall of below 500 millimetres (mm). The 2015/2016 El Niño drought was the worst drought Eswatini has experienced since 1992, costing in nominal monetary terms, an estimated $3.843 billion, representing 7.01% of Eswatini’s gross domestic product (GDP) in 2016 (SERPAC 2016). The drought in 1983 had the largest human loss of 500 people, whereas the 2005/2006 drought affected 410 000 people (EM-DAT 2018). With a population estimated at 1 403 362, it indicates that 13% of the population was affected, which is significant considering that the country is affected by many other hazards and burdens that include human immunodeficiency virus (HIV) and acquired and immunodeficiency syndrome (AIDS) (Central Intelligence Agency [CIA] 2018).

## Drought monitoring

In view of the impacts and increased frequency of drought in Eswatini, it is of critical importance to monitor droughts in near real-time and provide early warning information to stakeholders. Having reliable systems that detect the drought onset and monitor its development is an important step towards effective early warning systems. The definition and quantification of droughts are crucially important as they help in quantifying relief measures. The challenge, however, is the fragmentation of climatic data collection, the lack of agreed indicators and the poor coordination amongst institutions, thus resulting in drought management being rather reactive, or ‘crisis management’ approach, where the focus is on actions taken during, and shortly after a disaster, rather than before it happens. Therefore, the development of a comprehensive drought monitoring system capable of providing early warning of a drought’s onset, severity, persistence and spatial extent in a timely manner is a critical component in drought management (Chen et al. 2020; Hayes et al. 2011).

Traditional drought monitoring is generally based on drought indices that are computed from precipitation and remote sensing-based indices. By analysing and using a combination of indices, this research aims to develop an integrated framework for drought monitoring that incorporates standard precipitation index (SPI) and normalised difference vegetation index (NDVI). The framework allows a paradigm shift to a more risk reduction approach, enabling a cost-effective and timely response to drought. The recommendations and framework developed in the research are intended to support Eswatini in the development of its drought policy and disaster management plans.

Drought indices are mostly used to monitor drought conditions. The SPI is one of the most common precipitation-based indexes used to monitor drought as it presents a quick measure with minimal data requirements. In southern Africa, dependence on precipitation data alone might not be sufficient to monitor drought in all the areas, especially where data can be incomplete, unavailable, untimely and unreliable. Complementing therefore weather-based data with satellite imagery is essential to identify the spatial and temporal dimensions of drought, and to attain a complete, up-to-date, comprehensive coverage of drought conditions (García-León, Contreras & Hunink 2019; Peters et al. 2002; Wilhite & Pulwarty 2017). Normalised difference vegetation index is a remote sensing-based index using satellite imagery that measures vegetation conditions (Rouse et al. 1974). Several researches have denoted the positive correlation between NDVI and SPI. The use of a combination of NDVI and SPI indices provides more reliable results for drought monitoring than any single index in the research area (Al-Quraishi, Qader & Wu 2020; Dutta, Kundu & Patel 2013). Integrating the two indices therefore provides a near real-time indicator for drought, allowing planners to provide timely information for drought preparedness, mitigation and response planning, thereby helping to lower the eventual drought relief costs, protect food security and reduce the humanitarian impact on the population (Al-Hedny & Muhaimeed 2020; Chen et al. 2020; Han et al. 2019; Mlenga, Jordaan & Mandebvu 2019; Ozelkan, Chen & Ustundag 2016).

## Research design and methodology

This research involved a case study of the drought monitoring approaches used in Eswatini to ascertain the effectiveness as well as opportunities to improve quantification and coordination of drought monitoring. The general climatic characterisation of Eswatini is subtropical with wet hot summers (October–March) and cold dry winters (June–August). Mean annual rainfall ranges from 1500 mm in the northern Highveld to 500 mm in the southern Lowveld. The national long-term average rainfall is 788 mm per year. Precipitation varies considerably from year to year, which may either lead to periods of flash floods or drought. Mean annual temperature varies from 17 °C in the Highveld to 22 °C in the Lowveld (Government of Swaziland [GoS] 2013). These climatic conditions make the country very vulnerable to meteorological hazards, such as drought, floods, gusty winds and lightening, as well as epidemics during the wet and hot season. The Lowveld is the hottest and driest zone, and the most vulnerable to drought.

### Methodology for proactive drought quantification

**FIGURE 1:**
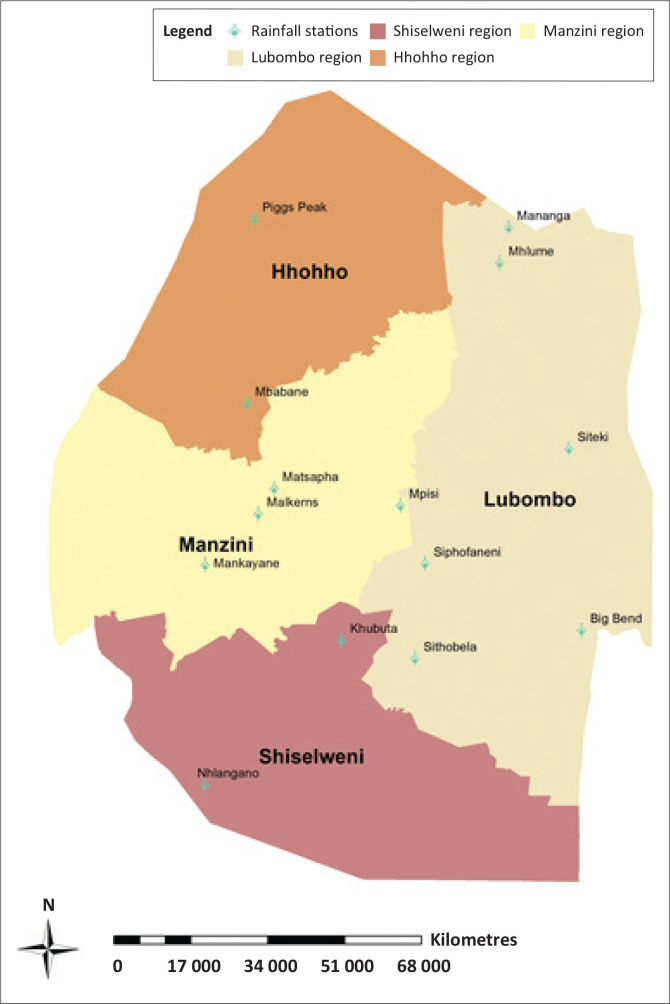
Map of Eswatini with rainfall stations used in the research.

The analysis of the temporal variation and frequency of droughts using a 3-month SPI (SPI-3) was conducted using DrinC software, using monthly rainfall data sets acquired for a period from 1986 to 2017 from 14 rainfall stations (Figure 1). Data sets were supplied by the Department of Meteorology and University of Eswatini. All the chosen weather (precipitation) stations exhibited good data quality by having minimal data gaps in the time series. The stations covered all agro-ecological regions and administrative regions in Eswatini. DrinC software was selected based on its simplicity, as it can be easily adopted for use. A series of at least 30 years period of data was used to determine the January, February, March, SPI-3values. The SPI-3 was selected because it is the period that coincided with the peak growing season. The primary data sources for NDVI were Climate Hazards Group InfraRed Precipitation with Station data (CHIRPS) is a 35+ year quasi-global rainfall data set produced by the Climate Hazards Group at the University of California, Santa Barbara and the Moderate Resolution Imaging Spectroradiomete (MODIS) Normalized Difference Vegetation Index Climate Modeling Grid (NDVI CMG) data made available by National Oceanic and Atmospheric Administration National Aeronautics and Space Administration (NOAA-NASA) and the NDVI based on Global Agricultural Monitoring (GLAM). The NDVI data in use were from the MODIS platforms Terra and Aqua, which have provided global coverage since 2000 (Terra) and mid-2002 (Aqua), at about 5 kilometres (km) resolution with a temporal frequency of overlapping 16-day periods.

The methodology for proactive drought determination was based on the statistical and threshold-based parameters from NDVI and time series of SPI-3. The World Meteorological Organization (WMO) recommends adopting the SPI to monitor the severity of drought events (Man-chi 2013), and Ji and Peters (2003) found that SPI-3 is the most effective for monitoring drought impact on vegetation, especially when the 3-month period coincides with the peak growing season. This is because the SPI-3 reflects short-term and medium-term moisture and provides a seasonal precipitation estimate (Ahmad et al. 2016; NDMC 2006; World Meteorological Organization [WMO] 2010). The relationship between NDVI and SPI was therefore investigated to determine how close the indices were in relation to explaining drought conditions. This was completed to ascertain whether their statistical relationship could be modelled to explain the occurrence and severity of a drought. The Pearson product–moment correlation coefficient, which is a measure of the extent of linear association between two variables and is represented by the value *R*, was used to explain the relationship between the two indices.

Using the formula below for calculating the correlation coefficient, the *R* value was calculated for the months of December (for the SPI) and January (for the NDVI) for the selected drought years:

r=Σ((X−My)(Y−Mx))/√((SSx)(SSy)) [Eqn 1]

The SPI-3, NDVI and temperature data from 2001 and 2017 were modelled to classify drought severity. Although SPI data were available from 1986, NDVI data were only available from 2001 onwards; therefore, only data for both indices from 2001 to 2017 were used for the regression analysis. The available data (Table 1) were used to develop a model to enable an early warning trigger threshold for droughts. The method used was the least squares, which is simply a minimisation of the sum of the squares of the deviations of the observed response from the fitted response (Naoum & Tsanis 2003). This involves the initial assumption that a certain type of relationship, linear in unknown parameters, holds. Drought severity is represented by the value of *Y* being the dependent (response) variable, and the model function is of a specified form that involves both the predictor variables (NDVI and SPI) and the parameters. Interaction effects between the variables were also considered. The unknown parameters were estimated under certain other assumptions with the help of available data, so that a fitted equation was obtained. In the model, drought determination was based on three main parameters: SPI, NDVI and temperature. The general form of the final model was:

Y=β0+β1 X1+β2 X2+β3 X3 [Eqn 2]

where

*Y* is drought occurrence (severity),

X1 is NDVI

X2 is SPI

X3 is the temperature.

**TABLE 1:** Normalised difference vegetation index, temperature, and standardised precipitation index for 2001–2017.

Year	NDVI	Temperature	SPI-3
2017	0.67	24.26	−1.54
2016	0.61	24.07	−1.90
2015	0.69	23.82	0.16
2014	0.72	23.93	0.72
2013	0.67	24.14	0.27
2012	0.68	24.23	−0.61
2011	0.72	23.99	1.35
2010	0.69	24.00	1.76
2009	0.66	23.22	−0.38
2008	0.69	23.39	0.45
2007	0.71	23.92	−0.44
2006	0.65	23.64	−0.48
2005	0.70	23.66	−0.28
2004	0.60	23.21	−0.63
2003	0.66	24.26	−1.08
2002	0.67	23.38	1.41
2001	0.67	23.45	0.81

NDVI, normalised difference vegetation index; SPI, standardised precipitation index.

### Development of drought monitoring and early warning framework

**FIGURE 2:**
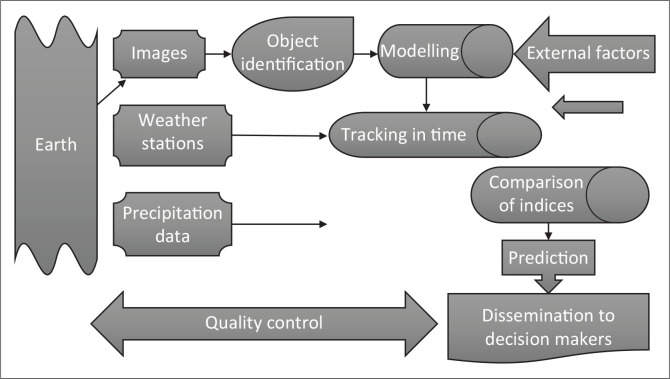
Artefact for the process of knowledge discovery from meteorological data and satellite imageries.

This research adopted Berhan et al.’s (2011) six research guidelines which include *identification, modelling, tracking, prediction, comparison,* and *communication with stakeholders.* The artefact for the process of knowledge discovery, which encompasses the six steps, incorporates use of data from satellite imagery as well as meteorological data. In the research, therefore, the artefact denotes the abstract representation (Figure 2) of the design-science research process and its communication to decision makers. The design theory, the artefact, its theoretical perspectives and the nature of its structure and processes involved were regarded as the main research tools and processes.

The artefact (framework) was designed to interact with a problem context in order to improve something in that context. To develop the artefact, the research looked at key elements necessary for an effective drought monitoring system in Eswatini. This included reviewing and collecting data on the following:

- key stakeholders
- drought knowledge
- drought management coordination
- drought monitoring and prediction
- drought communication and dissemination
- drought response capability.

To establish a comprehensive framework for drought monitoring, early warning and risk reduction in Eswatini, this research adopted the following steps.

### Data collection and processing

Rainfall, SPI and NDVI was determined through:

- Collection of precipitation and satellite imagery for the calculation of SPI and determination of NDVI.
- Collection of national or agro ecological zone rainfall data from representative meteorological stations with full data spanning over a period of 30 years or more.
- Calculation of SPI values using DrinC software.
- Collection of NDVI images from the GLAM - Global Agricultural Monitoring to determine NDVI values for the month of January.

### Drought quantification

Drought was quantified by the fitting SPI for the month of December and NDVI for the month of January into the following mode:

$$Y=\beta_{0}+\beta_{1}\;{\text{X}}_{1}+\beta_{2}\;{\text{X}}_{2}+\beta_{3}\;{\text{X}}_{3}$$ [Eqn 3]

where:

*Y* is the drought severity

X_1_ is NDVI

X_2_ is SPI

X_3_ is the temperature.

### Use of data from food security and vulnerability assessments

The research reviewed data from food security and vulnerability assessments such as Eswatini Vulnerability Assessment Committee (EVAC) report, Crop and Food Security Assessment Mission (CFSAM) or Integrated Food Security Phase Classification (IPC).

### Desk review and collection of secondary data

This research also involved literature review on previously related work in Eswatini, on agriculture, food security, environment policies and the impacts of droughts, NGOs and government drought interventions. Various data were collected from secondary sources, such as books, articles, local and national government reports, published interviews, newspaper clippings, mapping and diagramming. Because of the nature of this research, having multi-dimensional data sources, triangulation was used to negate or counterbalance the deficiency of a single data collection strategy so as to increase the validity and improve the ability to interpret the findings.

The research was built on the improvement of the existing tools to ensure the adoption and buy-in of the drought monitoring and early warning framework. The research analysed the weaknesses in the drought monitoring systems used in Eswatini and compared these with drought monitoring systems from southern Africa, Europe and Latin America. The research looked at country case studies for Botswana, India, South Africa, Zimbabwe and Uganda, from which the author identified the main mechanisms used in drought monitoring and management. The key regional early warning systems for Africa, Europe and Latin America were also reviewed to ascertain how they were linked to the national early warning systems, and the level and nature of support they provided. Based on the case studies the author conceptualised a drought monitoring and early warning framework incorporating results from modelling of SPI and NDVI, the role of stakeholders in monitoring and managing drought information.

### Developing a flowchart

The research defined the key elements, objectives and key stakeholders to be involved in the implementation, monitoring and funding of the system. Eswatini is utilising various pre- and post-drought monitoring tools. Key informant interviews were used to obtain information from key informants on their perceptions of drought monitoring and early warning. A total of 20 informants were selected through purposive sampling based on the organisations or institutions they worked for, and their expertise and involvement in the subject matter. The informants were drawn from international and local NGOs, UN, government ministries, district and provincial bodies, as well as the community and traditional leaders. Based on the information collected in steps 1–4, a concept was presented to stakeholders from which the framework was logically formulated in a participatory manner.

### Ethical consideration

This article followed all ethical standards for a research without direct contact with human or animal subjects.

## Results and discussion

### Correlation between normalised difference vegetation index and standardised precipitation index

**FIGURE 3:**
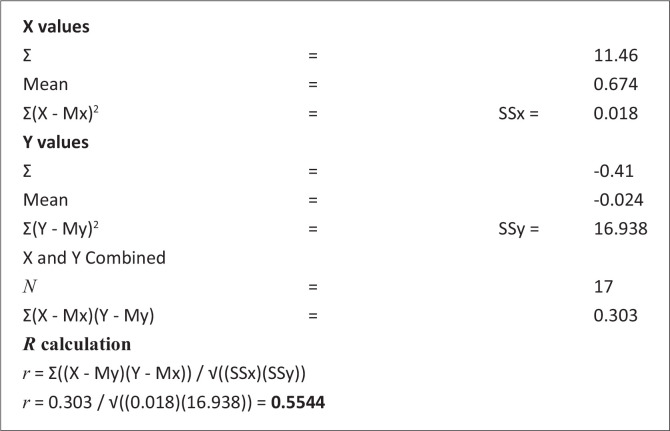
Pearson correlation coefficient between the normalised difference vegetation index and standardised precipitation index at standardised precipitation index-3 time scales.

**FIGURE 4:**
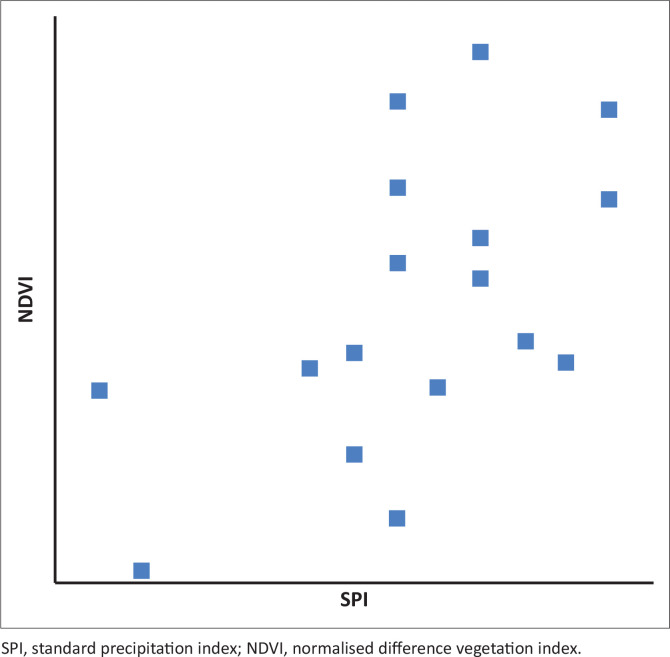
Correlation coefficient scatterplots for standardised precipitation index-3 time scale.

The *R*-value was calculated for the months of December (for SPI-3) and January (for NDVI) for the selected drought years. The results, values of *R* for SPI-3 and NDVI correlation coefficient, are presented in Figure 3, and the relationship is shown in the Figure 4 scatter plot. The correlation coefficient for both the SPI and NDVI for the time series 2002–2017 was *R*, determined at 0.55, which also demonstrated a positive correlation. This suggests that high *X* variable scores go with high *Y* variable scores (and vice versa). The scatter points are close to the line, indicating that the two variables had a positive correlation, indicating a moderate to positive linear relationship between the variables.

The NDVI for the month of March for all drought periods ranged between 0.60 and 0.72, with 0.60 being the year the country experienced an extremely pronounced El Niño weather system categorised as the worst El Niño to affect southern Africa in 35 years. The SPI-3 values ranged from 1.41 to −1.90. For the main drought years 2006/2007 and 2015/2016, the SPI-3 closely indicated a severe drought, whilst the NDVI was the lowest. Similarly, in the non-drought years for the same months, SPI values were mostly positive, whereas the NDVI was also high.

**FIGURE 5:**
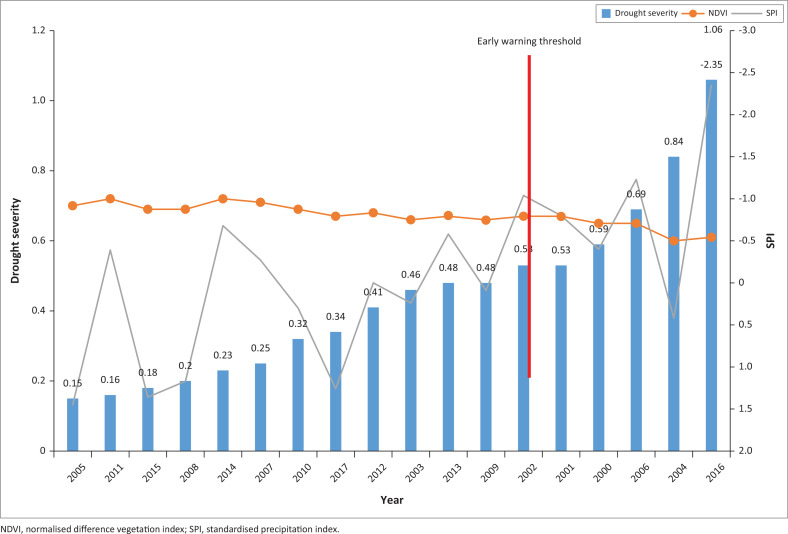
Determination of Y (drought severity).

Based on the model, integrating NDVI, SPI and temperature, the research determined the value of *Y* (drought severity). The value of *Y* varied between 0.06 and 0.84 for the years 2001–2017, with lower *Y* values indicating wet years and higher *Y* values representing drier years. However, not all high *Y* values represented drought years. There was a non-linear relationship between NDVI and SPI with intercepts at *Y* greater than 0.54 (Figure 5). Looking at the *Y* values, it was consistent that a value greater than 0.54 represented a significantly dry year, resulting in reduced cereal production, and in some cases, in an official drought declaration. It was noted that 50% of the time the Government of Eswatini officially declared a drought, *Y* was > 0.54 for SPI-3 time scale. Similarly, 100% of the time the *Y* value of > 0.54 was considered a dry spell or drought by government and UN and/or NGOs, based on reduced yields and increased vulnerably, as defined by Eswatini vulnerability assessment reports.

Determination of *Y* value through complementing weather and remote sensing data can help to provide a clearer picture of drought conditions. The combination of NDVI and SPI is important for enhancing the quality and reliability of drought information, as it provides better information on location and coverage of drought, which is useful for both planning and decision-making. There was a positive correlation between SPI and NDVI, indicating that SPI and NDVI – when used in combination – can be employed to detect the start of drought in Eswatini. The results of *Y* > 0.54 at 3-month time scale indicated that the country was experiencing a drought. The findings therefore confirm that SPI and NDVI, supported by other tools, are useful for assessing the extent and severity of drought. Consequently, the NDVI and SPI are adequate and proficient in providing a near real-time indicator of drought conditions at national level and across Agro-Ecological Zone.

### Drought monitoring and early warning framework

Countries in sub-Saharan Africa have climate information and prediction services, which are normally government-managed meteorological services, and services provided by research institutions and universities. However, the challenge is that the collection of data is usually poorly coordinated between agencies and ministries because of government structures in most of the countries (Wilhite 2006). The key challenges are inadequate infrastructure and instruments, lack of qualified staff and ineffective dissemination facilities (SADC 2012; Sigudla 2015). A review of the global, national and regional drought monitoring tools has shown that there are a variety of tools used (Table 2). Moreover, there are also differences amongst the drought indicators adopted. The common drought indicators used were mostly SPI, rainfall data and the NDVI. Consequently, the use of both SPI and NDVI in this research is consistent with the drought indicators adopted by various countries in Africa, Europe, America and Asia.

**TABLE 2:** Global and national drought monitoring products.

Coverage	Drought product	Drought indicator
Global	Flood and Drought Portal	Standardised precipitation index
		Effective drought index
		Normalised difference vegetation index
		Vegetation condition index
		Soil water index
		Vegetation health index
		Agricultural stress index
		Combined drought index
Global	FAO’s Agricultural Stress Index System	Agricultural stress index
Africa	African Flood and Drought Monitor	Standardized precipitation index
		Normalised difference vegetation index
		Drought index
		Streamflow percentile
South Africa	National Integrated Water Information System – Drought status and management	Dam levels
		Rainfall
		Standardised precipitation index
Turkey	Meteorological Drought Situation according to standardised precipitation index method	Standardised precipitation index
UK	UK Drought Portal	Standardised precipitation index
US	United States Drought Monitor	Palmer drought severity index
		CPC Soil Moisture Model (percentiles)
		USGS Weekly Streamflow (percentiles)
		Standardised Precipitation Index
		Objective drought indicator blends (percentiles)
India	Drought Monitoring	Aridity Anomaly Index
		Standardised Precipitation Index

*Source:* World Meteorological Organization (WMO) and Global Water Partnership (GWP), 2018, *The three pillars/monitoring & early warning. Monitoring & early warning*, viewed 24 October 2018, from http://www.droughtmanagement.info/pillars/monitoring-early-warning/.

FAO, Food and Agriculture Organization of the United Nations; UK, United Kingdom; US, United States.

A review of the different country and regional drought monitoring systems (Tables 2 and 3), analysing the differences and commonalities, provided a basis for conceptualising a proactive risk management strategy for Eswatini. From the analysis of different case studies, it was noted that establishing a proactive risk management strategy requires the use of information provided from reliable drought monitoring tools. By using this set of data, the risk of drought and its impact on food security is analysed in real time. Although the current systems for early warning and drought control relatively function, some challenges or gaps need to be addressed to improve the efficacy and efficiency of the existing systems. Commonalities of all country and regional drought monitoring and early warning systems that were also adopted for Eswatini were the following:

- Drought monitoring and prediction:
  ■ Drought monitoring using drought indices and remote sensing.
  ■ Use of drought indices is complemented with food security, agriculture and vulnerability assessments.
- Drought communication and dissemination:
  ■ Decentralised drought declarations. Declarations can be made at national level or sub-national level.
  ■ Differences in time and the timeliness of declaration from state to state.
  ■ For a system to work, there is a need of an effective information management system.
  ■ Use of various media channels for communication.
  ■ There is a need to develop policies to support the functioning of the system.
  ■ There is a need for releasing monthly or quarterly agro-met bulletins.
- Response capability/coordination
  ■ There should be a strong network of stakeholders and organisations dealing with drought monitoring and mitigation. This involves different ministries, research and private sector.
  ■ There is a need for response plans in place for effective drought response.
- System challenges
  ■ Most monitoring is implemented when the drought’s impact is being felt.

**TABLE 3:** Review of country and regional drought monitoring systems.

Country level	Key features
Botswana	A drought and household food security outlook tour is undertaken annually, after the rainy season (April–May) to complement early warning reports compiled on a routine basis by the various government departments and ministries (Manthe-Tsuaneng 2014; UNW-DPC 2015).
South Africa	The NDMC is responsible for classifying drought as a natural disaster and consults with provincial disaster management centres, and assesses the magnitude and severity of the ongoing drought condition in the country. A classification of a national disaster ensures the national executive has the responsibility to coordinate and manage the disaster in close cooperation with provincial and local government, the private sector and civil society, using the applicable contingency plans and existing legislative mechanisms at its disposal to deal with the effects of the disaster effectively (CoGTA 2018; NDMC 2018).
Zimbabwe	There are two ministries that carry out drought monitoring in Zimbabwe: the Ministry of Environment, Water and Climate (through the Meteorological Services Department) and the Ministry of Agriculture (through Agriculture Research and Extension Services). The roles and responsibilities of the two departments include systematic observation and monitoring of hydro-meteorological parameters, the publication of information and forecasts, and the provision of products and services related to weather and climate (Mavhura 2017; Nangombe 2014).
Uganda	The Uganda DEWS consists of collecting data on a monthly basis from communities, district offices and the Department of Meteorology, analysing it at district level in collaboration with district heads of department, producing a monthly drought bulletin and disseminating key messages to communities and development partners (Atyang 2014).
**Regional level**	
Africa	The Intergovernmental Authority on Drought and Development (IGAD) Climate Prediction and Applications Centre (ICPAC) consists of a network of national meteorological and hydrological services from 11 countries in total in the Greater Horn of Africa. These are Djibouti, Eritrea, Ethiopia, Kenya, Somalia, Sudan, South Sudan, Uganda (all member countries) together with Burundi, Rwanda and Tanzania. The network develops early warning products and organises forums, bringing them together with information users (ICPAC 2018).
	The Famine Early Warning Systems Network (FEWS NET) is a USAID-funded activity that works together with international, national and regional partners to provide timely and rigorous early warning, and vulnerability information on emerging and evolving food security issues. Furthermore, FEWS NET monitors and analyses relevant data and information in terms of the impact on livelihoods and markets to identify potential threats to food security (FEWS NET 2018).
	The Southern African Regional Climate Outlook Forum (SARCOF) is a regional, seasonal weather outlook prediction and application process adopted for the SADC. The process facilitates information exchange amongst forecasters, decision-makers and climate information users. Its main objective is to promote technical and scientific capacity-building in the region by producing, disseminating and applying climate forecast information in weather-sensitive sectors of the region’s economic activities (SADC 2018; WMO/GWP 2018).
	The SADC Climate Services Centre (CSC) contributes to the mitigation of adverse impacts of extreme climate variations on socio-economic development, by monitoring near real-time climatic trends and generating medium-range (10–14 days) and long-range climate outlook products, on monthly and seasonal (3–6 months) timescales (SADC 2018).

*Source:* World Meteorological Organization (WMO) and Global Water Partnership (GWP), 2018, *The three pillars/monitoring & early warning. Monitoring & early warning*, viewed 24 October 2018, from http://www.droughtmanagement.info/pillars/monitoring-early-warning

Looking at the key challenges of the current monitoring and preparedness mechanisms mentioned during interviews with stakeholders from different government ministries, research institutions and Tinkhundla administration, it was apparent that there were coordination challenges amongst drought monitoring agencies. Of the stakeholders interviewed, only 42% felt that capabilities of Eswatini to monitor drought were efficient, despite the existence of the National Disaster Management Agency, which coordinates response mechanisms, and the Ministry of Agriculture coordinating drought monitoring with other agencies. The respondents mentioned that the coordination structure needs to be further improved.

Coordination, in this research, was considered as a key element that allows bringing together various stakeholders into a common forum for effective monitoring of drought, and for providing effective and harmonised communication on early warning products. Stakeholders also highlighted the need for increased resources, improved tools for drought monitoring and capacity development. Of concern to the key informants was the timeliness of drought information products and declarations. The field-level data collected by the Ministry of Agriculture only happens when drought impacts are being felt. Thus, the response is more reactive than proactive. Although the information is useful for planning and response, the majority of the respondents believe that the assessments are late to enable effective preparedness mechanisms. From these factors, it was shown that the current system needs some improvements.

**FIGURE 6:**
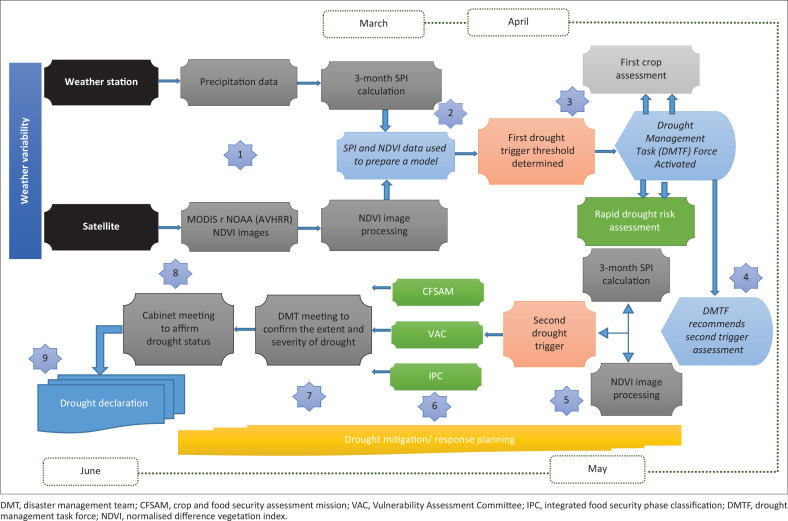
Drought monitoring and early warning framework.

Putting together the various elements that include drought indices, modelling and various case studies, the following drought monitoring and early warning framework (Figure 6) was conceptualised and proposed for Eswatini. The drought monitoring and early warning framework was based on the following elements:

- drought knowledge
- drought management coordination.
- drought monitoring and prediction
- drought communication and dissemination
- response capability.

The drought monitoring and early warning framework incorporates the following steps:

- Data collection and processing

Collection of precipitation and satellite imagery for the calculation of SPI and determination of NDVI.

- Drought quantification – Level 1

Using SPI for the month of December and NDVI for the month of January to determine drought severity using the mode *Y* = β_0_ +β_1_ X_1_ + β_2_ X_2_ + β_3_ X_3_. For *Y* that is greater than 0.54 indicates a significant dry spell that is likely to result in reduced cereal yields. With *Y* > 0.54 a drought Management Task Force (DMTF) is activated to coordinate the drought management process. Based on the value of *Y*, the DMTF decides whether additional data is required to ascertain the severity and extent of the drought. Crop assessment and other rapid vulnerability assessments can be imitated to verify the drought status.

- Drought management task force review

Based on the results of the vulnerability assessments, the DMTF can recommend a second drought quantification. If the assessment results have confirmed the extent and severity of the drought, Step 5 can be omitted and recommendations for a CFSAM, EVAC or IPC can be made to determine the extent of aid or support the country will require.

- Drought quantification – Level 2

Using SPI-3 for the month of April and NDVI for the month of May, they are fitted into the model determined in Step 2. For a value of *Y* greater than 0.54 indicates a significant dry spell and recommendations for a CFSAM, VAC or IPC can be made to determine the extent of aid or support the country will require. The step will characterise dry spell in levels that will trigger different responses. Drought mitigation/response planning is initiated to ensure that when a declaration is made all state apparatus is ready for a response. Other stakeholders, such as NGOs and the UN, can also start their own planning and resource mobilisation.

- Conducting food security and vulnerability assessments – Eswatini Vulnerability Assessment Committee, Crop and Food Security Assessment Mission **or** Integrated Food Security Phase Classification

The DMTF will coordinate the administration of any of the standard food security and vulnerability assessments: SVAC, CFSAM or IPC. These assessments will be coordinated with various stakeholders, UN, NGOs, private sector and communities. The objective of these assessments is to validate the outcomes of the drought quantification process based on modelling.

- Drought management task force final review

A final DMTF meeting to confirm the extent and severity of drought is conducted and recommendations to government are prepared. A meeting is convened with cabinet within 1 week of technical drought determination to recommend official drought declaration, or drought response without official drought declaration.

- Government drought declaration

The government will officially declare a drought and put in place emergency measures to prevent a drought disaster.

## Institutional framework for drought monitoring and management

**FIGURE 7:**
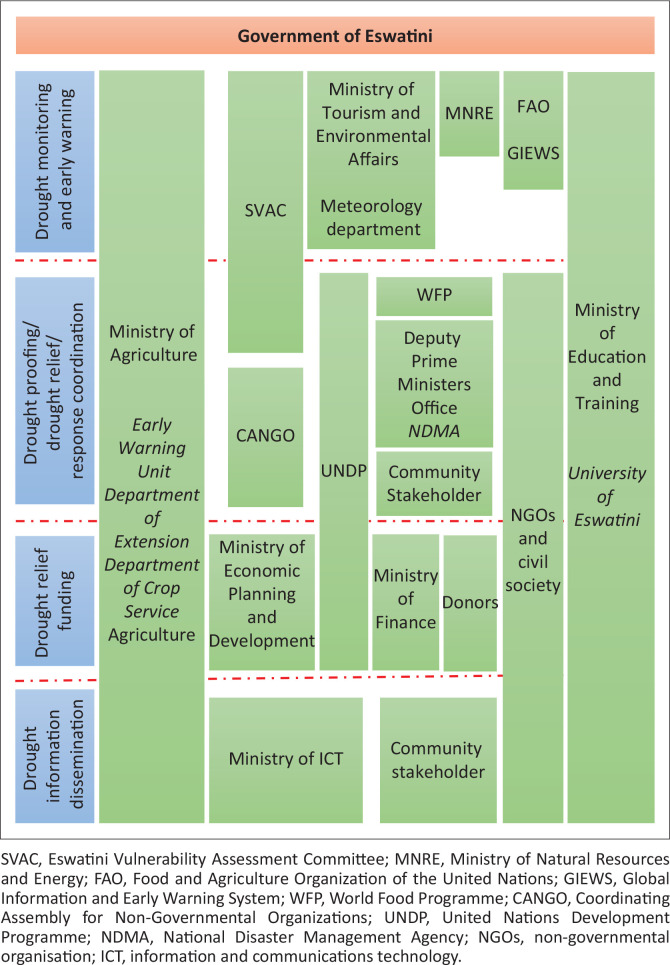
Institutional framework for drought monitoring and management.

Drought monitoring and management requires a strong institutional structure to monitor and provide a timely response to drought. To support the implementation of the drought monitoring and early warning framework, this research developed an institutional framework for drought monitoring and management (Figure 7). This institutional framework allows for an effective coordination at different levels of both the institutional framework and drought and early warning framework. This ensures that stakeholders understand their role at each stage in the monitoring and response continuum. The adoption of the drought monitoring framework and its mainstreaming into national development frameworks need to be a participatory process involving a variety of stakeholders, such as national and local governments, community-based and civil society organisations, research and scientific community, private sector and the media. The main government stakeholders include the Ministries of Foreign Affairs, Local Government, Natural Resources and Energy, Tourism, Environment and Communication, Economic Planning and Development, Tinkhundla, Health and Social Welfare, Public Works and Transport, Agriculture and Cooperatives and Finance and Disaster Management.

The proposed framework addresses many shortcomings of the current systems and country and regional processes, thereby improving the drought preparedness. Key issues that are addressed are presented in Table 4.

**TABLE 4:** Practical contributions of institutional framework for drought monitoring and management to existing drought monitoring mechanisms in Eswatini.

Key issues	Description
Continuous drought monitoring	Developing a continuous drought monitoring system will allow the enactment of national drought policies that focus on risk reduction, and the use of tools that provide early warning information. The task of drought monitoring and early warning will be the shared responsibility of many stakeholders and the framework will allow effective coordination of the process.
Integrated drought monitoring and early warning system	The framework introduces an operational drought monitoring and early warning prototype with well-connected sharing of information with various stakeholders, scientists, communities at different levels within the country and the region. The adoption of an integrated framework allows early buy-in of key decision-makers of early warning information, thereby enabling the government and its partners to enact mitigation and response planning mechanisms.
Effective spatial drought monitoring	The framework allows the use of vegetation health indicators and rainfall anomalies from satellite data and targeted *in situ* data collection vulnerability and crop-yield data. The use of NDVI and SPI therefore enables the country to implement drought risk analysis across agro-ecological regions as well as prepare region or district-specific adaptation measures.
Effective communication	The framework will serve as a link between drought task force and various stakeholders by communicating timely information to all stakeholders concerned with drought and its impacts. Effective communication will be achieved through dissemination of customised national drought monitoring products both through centralised at government level and decentralised at regional and community levels.
Funding	The framework acknowledges the need to have a sustained funding model to enable the drought monitoring and early warning system to function. The involvement of the Ministry of Finance in the framework allows the incorporation of the framework in the government budget planning and objective setting.
Effective drought management coordination	The framework allows for effective coordination and prioritisation of activities and response. By virtue of having a task team task force and coordination mechanisms at different levels allows bringing ideas and issues together on drought and its potential impacts on the table, thereby allowing timely prioritisation and decision-making.

NDVI, normalised difference vegetation index; SPI, standard precipitation index.

## Conclusion

Drought magnitude, duration and intensity in Eswatini are increasing over time. The spatiotemporal analysis of drought is of great importance to Eswatini as the country has been facing recurring droughts with negative impacts on agriculture, environment and economy. The monitoring, prediction, management and mitigation of the drought hazard are essential elements for reducing the impact of droughts on the Eswatini population. The gathering of climatic data is however fragmented between government agencies in Eswatini; there is limited use of multiple indices being used to explain the drought hazard, and there is no coordinated mechanism in place to collect, monitor and communicate drought information in a proactive rather than a reactive manner. This research identified the lack of drought policy frameworks and poor coordination between institutions that provide drought early warning information as the main hindrances of having an effective drought disaster management plan.

To counter these limitations, this research developed an integrated drought monitoring and early warning framework that encompasses effective coordination and use of precipitation and remote sensing-based drought indices. The framework adopts an integrated and novel approach for monitoring and assessment of the drought risk based on a combination of meteorological data, NDVI from satellite imagery and targeted collection of ground truth, crop-yield and vulnerability data. The SPI-3 computation and use of NDVI detected the onset of early-season drought, thereby affirming the applicability of drought indices for monitoring near real-time and retrospective droughts in Eswatini. The combined use of spatiotemporal information from both NDVI and SPI time scales allowed for the effective monitoring of the onset, severity and magnitude of droughts. The conceptualisation of the operational drought monitoring and early warning prototype, with well-connected sharing of information with various stakeholders, scientists and communities at different levels within the country and the region, allows early buy-in of key decision-makers of early warning information, thereby enabling the government and the UN agencies to enact mitigation and response planning mechanisms.

The framework addresses shortcomings in the existing drought monitoring mechanisms in Eswatini by ensuring the availability of accurate, reliable and high-resolution characterisations through objective science-based methods, information that can be used to make drought preparedness, mitigation and response decisions by stakeholders and communities. The framework facilitates informed drought declarations and enables the dissemination of reliable and timely early warning information for use by governments and stakeholders to identify periods of enhanced risk and to trigger assistance measures.

## Recommendations

The framework proposed mainly centres on the operational aspect of monitoring and does not dwell on other facets of the disaster management cycle, especially drought planning, response and recovery. Legislation to ensure that drought risk reduction strategies are implemented should be developed and enforced. Moreover, emphasis should be placed on the prevention, mitigation and preparedness rather than rely solely on crisis management. The drought monitoring and early warning framework should be further evaluated and compared with other drought monitoring tools with the involvement of regional researchers, governmental authorities and policymakers so as to allow a harmonised approach in the region for drought monitoring and sharing harmonised drought-related products.
